# Comparison of Chemoport Implantation in Interventional Radiology Suite vs. Operating Room: Focus on Complications

**DOI:** 10.3390/medicina62050962

**Published:** 2026-05-14

**Authors:** Young-Heun Shin, In Chul Nam, Doo Ri Kim

**Affiliations:** 1 Department of Surgery, Jeju National University Hospital, School of Medicine, Jeju National University, 15, Aran 13-gil, Jeju 63241, Republic of Korea; youngheunshin@gmail.com; 2 Department of Radiology, Jeju National University Hospital, School of Medicine, Jeju National University, 15, Aran 13-gil, Jeju 63241, Republic of Korea

**Keywords:** chemoport, intervention, radiology, surgery, operating room, complications

## Abstract

*Background and Objectives*: Chemoports are essential for long-term chemotherapy. They are typically inserted either by interventional radiologists in an interventional radiology (IR) suite or by surgeons in the operating room (OR). Although both approaches are widely used, it remains unclear whether the procedural setting affects complication rates. In this study, we compared the safety of chemoport implantation in IR and OR and evaluated the impact of patient-related factors. *Materials and Methods*: We retrospectively reviewed 737 adult patients who underwent chemoport placement between January 2021 and December 2023 at a single tertiary institution. Of these, 544 ports were inserted into IR suites and 193 in ORs. All IR procedures used fluoroscopic and ultrasound guidance, whereas OR procedures used ultrasound without intraoperative fluoroscopy. Complications were classified as early (≤30 d) or late (>30 d). Kaplan–Meier analysis and Cox regression analysis were used to assess complication-free survival and identify independent predictors. *Results*: A total of 45 complications (6.1%) occurred, including 25 infections (3.4%), 9 wound dehiscences (1.2%), and 11 malfunctions (1.5%). There were no significant differences in overall, early, or late complication rates between the IR and OR groups (*p* > 0.05). Kaplan–Meier analysis demonstrated comparable complication-free survival between the groups (log-rank *p* = 0.285). Cox regression analysis identified no independent predictors of complications, although higher BMI showed a borderline association (adjusted HR, 1.076; 95% CI, 1.000–1.158; *p* = 0.051). *Conclusions*: Chemoport implantation performed in IR suites is as safe as that in ORs, with similar early and late complication rates.

## 1. Introduction

Chemoports provide reliable and durable central venous access for patients requiring long-term chemotherapy. Chemoport implantation is typically performed either by interventional radiologists in an interventional radiology (IR) suite or by surgeons in an operating room (OR) [1,2,3,4,5]. Both approaches aim to achieve safe catheter placement, most commonly via the internal jugular vein (IJV), but differ in procedural environment and imaging guidance. In IR suites, procedures are performed under fluoroscopic and ultrasound guidance, enabling real-time visualization of guidewire and catheter placement. In contrast, in OR settings, surgeons often use ultrasound for venous puncture but may omit intraoperative fluoroscopy, confirming catheter tip position postoperatively via chest radiography [6]. These differences raise the question of whether the procedural setting influences complication rates.

Previous studies have reported low complication rates (approximately 3–10%) and found no significant differences between IR- and OR-based insertions [6,7]. Park et al. compared 375 chemoport implantations (203 IR vs. 172 OR) and reported no significant differences in complication type or rate between radiologist- and surgeon-placed ports [7]. Similarly, larger retrospective analyses have demonstrated equivalent safety outcomes regardless of procedural setting, with comparable rates of infection, mechanical malfunction, and thrombosis [6]. Several studies have also suggested that patient-related factors such as diabetes mellitus (DM) and body mass index (BMI) may be associated with chemoport-related complications, although evidence remains inconsistent [8,9,10,11].

Therefore, in this retrospective study, we aimed to compare the complication profiles of chemoport insertions performed in the IR suite versus the OR and to evaluate whether DM, BMI, and the choice of right or left IJV access influence complication risk.

## 2. Materials and Methods

This study was approved by our Institutional Review Board (IRB No.: JEJUNUH 2025-07-016). The requirement for informed consent was waived due to the retrospective nature of the study.

We retrospectively reviewed adult patients who underwent chemoport insertion between 1 January 2021 and 31 December 2023 at our institution. Initially, 740 patients were identified. We excluded 1 patient who died within 30 d of port placement (unrelated early mortality) and 2 patients whose ports were inserted via the femoral vein, resulting in a final cohort of 737 patients. Of these, 544 ports were placed in the IR suite by interventional radiologists, and 193 were placed in the OR by surgeons.

In the IR group, all IR procedures were performed under local anesthesia with full imaging guidance. Ultrasound was used for the IJV puncture, and fluoroscopy was used for guidewire navigation, catheter placement, and confirmation of the catheter tip in the superior vena cava.

In the OR group, ultrasound guidance was used for venous access (internal jugular puncture), and all procedures were performed under anesthesia in a sterile surgical field. Unlike the IR group, intraoperative fluoroscopy was not routinely employed in the OR; instead, surgeons relied on measurements and postoperative chest radiography to verify catheter tip position.

**Figure 1:**
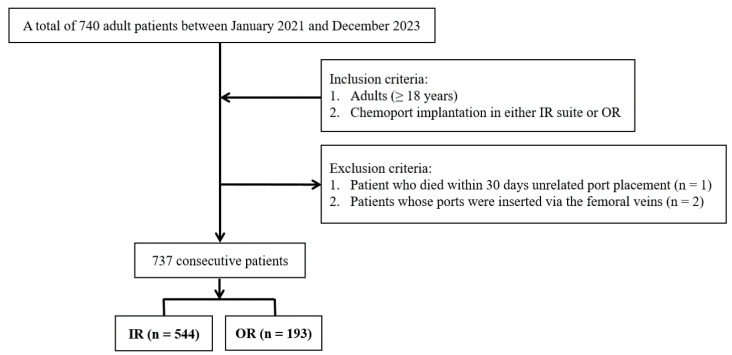
Case accrual process.

The right IJV was the preferred access site in both groups. The left IJV was used when right-sided access was not feasible or contraindicated (e.g., right-sided breast cancer or venous occlusion). All patients received prophylactic antibiotics (first-generation cephalosporins) before port insertion, and the ports were handled using a standard sterile technique during access. Figure 1 illustrates the patient inclusion and exclusion process.

### 2.1. Data Collection

We recorded patient demographics and potential risk factors, including age, sex, DM status, and BMI. Complications associated with chemoport were classified as early (≤30 d of the procedure) or late (>30 d post-insertion) based on established definitions [12]. Complications of primary interest included infection, wound dehiscence, and port malfunction. Infections encompassed both port-pocket and catheter-related bloodstream infections, identified based on local (erythema, warmth, purulent discharge at the port site) or systemic (fever, chills, positive blood cultures) signs, following definitions from prior studies [13]. Confirmed infections led to port removal and targeted antibiotic therapy. Wound dehiscence was defined as the separation of the incision or port-pocket wound, sometimes resulting in port exposure. Port malfunction included mechanical problems such as catheter occlusion (inability to flush or draw blood), thrombosis, catheter malposition or dislodgement, and port reservoir issues (e.g., flipping or rotation of the port, making access impossible). Other potential complications, such as pulmonary embolism, pneumothorax, hematoma, or arterial injury, were also monitored, although none were significant in our initial data review.

### 2.2. Follow-Up

Patients were followed from the time of port insertion until 28 February 2025, or until an event of interest (port removal due to complications) or death, whichever occurred first. This provided a maximum potential follow-up period of approximately 50 months for the earliest cases. For patients without complications, follow-up endpoints included completion of chemotherapy (planned port removal at the end of treatment) or the end of the observation period with the port still in place, in which case they were censored at the last known follow-up. Mortality within 30 d was extremely low (only one case was excluded because it was unrelated to the port), and long-term mortality was attributable to the underlying disease rather than the port itself. Patients who died with a functioning port in situ and without port-related complications were censored at the time of death for the complication-free survival analysis.

### 2.3. Statistical Analysis

Categorical variables were compared using the chi-square test or Fisher’s exact test. Normality of continuous variables was assessed using the Shapiro–Wilk test. Continuous variables were analyzed using the Mann–Whitney U test due to non-normal distribution. Kaplan–Meier survival analysis was performed to compare complication-free survival between the two groups, and the log-rank test was used to compare survival curves. Finally, a Cox proportional hazards regression model was applied to adjust for confounding factors and identify independent predictors of complications. All statistical analyses were conducted using SPSS software (version 22; IBM Corp., Armonk, NY, USA).

## 3. Results

This study included 399 men (54.1%) and 338 women (45.9%) with a mean age of 67.8 ± 12.6 years. The IR group included 544 patients (mean age, 67.6 ± 12.4 years), comprising 283 (52%) men and 261 (48%) women. The OR group included 193 patients (mean age, 68.6 ± 13.1 years), comprising 116 (60.1%) men and 77 (39.9%) women. DM was present in 134 patients (18.2%), including 96 (17.6%) in the IR group and 38 (19.7%) in the OR group. The mean BMI was 22.8 ± 4.3 kg/m^2^, with no significant difference between the IR (22.9 ± 4.3 kg/m^2^) and OR (22.6 ± 4.1 kg/m^2^) groups. The right IJV was predominantly used as the access site in both groups. In the IR group (*n* = 544), 468 procedures (86.0%) were performed on the right side and 76 (14.0%) on the left. In the OR group (*n* = 193), the right side was used in 179 patients (92.7%) and the left in 14 (7.3%). Overall, the right side accounted for 647 cases (87.8%) and the left for 90 (12.2%) among the total study population (*n* = 737). All insertions were successful on the first attempt. No immediate procedure-related major complications, such as pneumothorax, arterial puncture, or severe hematoma, were observed. Table 1 summarizes patient demographics.

A total of 45 complications occurred during follow-up. Among these, 25 (55.6%) were infections, 11 (24.4%) were malfunctions, and 9 (20.0%) were cases of wound dehiscence. Early complications (≤30 d) occurred in 2 patients (4.4%)–one in each group–both of whom presented with infection. Specifically, the IR case was a bloodstream infection, whereas the OR case was a port site infection; both required port removal. Late complications (>30 d) were observed in 43 patients (95.6%): 30 in the IR group and 13 in the OR group. Of these, 25 had infections, 9 had wound dehiscence, and 11 had wound malfunction. Table 2 summarizes complications between two groups.

**Table 2:** Comparison of complications between the IR and OR groups.

	IR (*n* = 544)	OR (*n* = 193)	Total (*n* = 737)	*p* Value
Overall complication	31 (5.7%)	14 (7.3%)	45 (6.1%)	0.484
Early complication (<30 d)	1 (0.2%)	1 (0.5%)	2 (0.3%)	0.443
Late complication (>30 d)	30 (5.5%)	13 (6.7%)	43 (5.8%)	0.534
Infection	20 (3.7%)	5 (2.6%)	25 (3.4%)	0.107
Wound dehiscence	5 (0.9%)	4 (2.1%)	9 (1.2%)	0.428
Malfunction	6 (1.1%)	5 (2.6%)	11 (1.5%)	0.277

**Figure 2:**
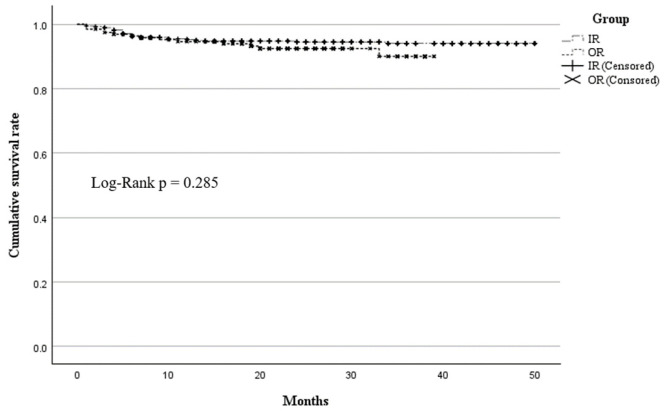
Kaplan–Meier curve showing cumulative complication-free survival of the chemoport implantation between IR and OR.

Analysis of patient characteristics revealed no statistically significant differences in the incidence of complications according to age, sex, treatment group (IR vs. OR), DM status, BMI, or venous access approach (right vs. left IJV). The incidence of total complications did not differ significantly between patients with and without DM (*p* = 0.195). We constructed Kaplan–Meier curves for complication-free survival of port insertion between IR and OR (Figure 2). At 12 months, the estimated complication-free survival rates were 95.2% and 94.6% in the IR and OR groups, respectively. At 24 months, the overall survival was 94.5% in the IR group and 92.4% in the OR group. The curves were closely aligned up to approximately 34–35 months (94% in IR and 90% in OR). Beyond this point, the number of patients at risk decreased substantially, and after approximately 40 months, only one group remained due to censoring, limiting further comparison. Although the IR curve was slightly higher, the log-rank test showed no statistically significant difference (*p* = 0.285). In both univariate and multivariate Cox regression analyses, no variables were statistically significant predictors of complications. However, BMI showed a marginal trend, with each unit increase associated with a 7.6% higher risk of complications (hazard ratio [HR], 1.076; 95% confidence interval [CI], 1.000–1.158; *p* = 0.051). Other factors, including treatment group, sex, age, DM, and venous access approach, were not significantly associated with complication risk. Table 3 summarizes the results of the univariate and multivariate Cox proportional hazards analyses.

## 4. Discussion

Our retrospective study demonstrated that chemoport implantation performed in an IR suite is as safe as that performed in an OR in terms of both 30-day and long-term complication rates. There was no statistically significant difference in the incidence of complications–including infection, wound dehiscence, and mechanical malfunction–between the two settings. This finding agrees with previous research [7], reinforcing the notion that when proper sterile techniques and ultrasound guidance are used, the procedural setting (radiology vs. surgery) does not intrinsically affect patient outcomes. Notably, Park et al. also concluded that chemoport implantation can be safely performed in either the OR or IR without differences in complication rates [7]. Our results support these findings in a larger cohort than that of the previous study, with 737 patients in total, including 544 in the IR group and 193 in the OR group.

One practical distinction in our series was the use of fluoroscopy during the procedure in the IR setting, whereas it was not used in the OR. However, we did not observe a significant increase in malpositioning or early malfunction in the OR group. This suggests that careful measurement and routine post-procedural radiography in the OR are sufficient to ensure proper tip placement in most cases. Real-time fluoroscopy allows immediate detection of misplacements; thus, one might expect fewer misplacement-related complications in IR procedures. Indeed, the literature supports the idea that radiologic guidance minimizes malpositioning [9]. The comparable outcomes in our study highlight that experienced operators can mitigate this difference. From a cost and efficiency perspective, however, avoiding additional relocation procedures—or operative “guess-and-check” steps—is advantageous, giving IR a potential cost benefit. Prior cost analyses strongly favored IR [14]; LaRoy et al. reported that the average cost of OR placement was nearly double that of IR, and a more recent study by Martin et al. found a per-patient cost savings of approximately $1170 with IR placement [6]. In our context, performing port insertions in the IR suite may free up OR resources and reduce overall hospital costs without compromising care quality. This is particularly relevant in high-volume cancer centers, where hundreds of ports are located annually.

The observed complication profiles provide important clinical insights. Infections remained the leading cause of port failure in our cohort, consistent with other studies [13,15]. The IR group showed a slightly higher absolute number of infections; however, this difference was not statistically significant. Nevertheless, our infection rates (IR, 3.7%; OR, 2.6%) were comparable to those reported in a previous study [13]. Maintaining strict sterile techniques during insertion and ensuring meticulous maintenance by trained nursing staff are critical for keeping low infection rates. Wound dehiscence was an infrequent complication (1.2% overall); however, it underscores the importance of a meticulous surgical technique for port pocket creation and incision closure. In our comparison, the OR group had a slightly higher rate of wound issues (2.1% vs. 0.9%), although this difference was not statistically significant. This could be related to differences in closure materials or techniques (e.g., surgeons might use larger incisions or vary in their preference for sutures versus glue). IR physicians typically make smaller incisions sufficient for the port and use subcuticular sutures with tissue adhesives. Ultimately, preventing dehiscence also helps prevent infection, as early infection can impair wound healing. We recorded 11 cases (1.5%) of port malfunction unrelated to infection, including catheter occlusion (suspected thrombosis or fibrin sheath formation) and mechanical failure. Specifically, six malfunctions occurred in the IR group (1.1%) and five in the OR group (2.6%). Malfunctions tended to occur later (median 5.7 months, 1.2–34.3 months post-insertion), typically after prolonged use. IR fluoroscopic placement likely ensured optimal initial positioning, reducing the risk of catheter tip thrombosis or kinking, whereas in a few OR cases, tip positioning may have been initially suboptimal, even if later confirmed radiographically.

In both univariate and multivariate analyses, BMI did not show a statistically significant independent effect, although a higher BMI showed a trend toward increased risk of complications (hazard ratio [HR], 1.076; 95% confidence interval [CI], 1.000–1.158; *p* = 0.051). This aligns with mixed findings in the literature. One retrospective study reported that BMI > 28.75 was associated with higher overall complication rates [11], whereas a larger prospective study of 815 patients found no significant association [10]. Further large-scale studies are warranted to clarify the relationship between BMI and chemoport-related complications.

Similarly, DM was not significantly associated with chemoport-related complications in either univariate or multivariate analyses. Although DM is generally considered a risk factor for infection and impaired wound healing [8], this relationship was not evident in our cohort. The incidence of total complications did not differ significantly between patients with and without DM (*p* = 0.195). However, our analysis was limited by the binary classification of DM status. We assessed only the presence or absence of DM without accounting for factors such as the degree of glycemic control, disease duration, treatment status, or diabetic complications. These unmeasured factors may have influenced outcomes and potentially introduced residual bias. A multicenter study of 1714 port placements identified DM as a significant risk factor for early port infection (within 30 d), with patients with DM having an approximately 3.7-fold higher infection risk [8]. Future studies incorporating detailed metabolic parameters (e.g., HbA1c levels), DM duration, and comorbidity profiles are needed to better elucidate the impact of diabetes on chemoport-related complications.

Our study had some limitations. First, it was a single-center retrospective study, inherently susceptible to selection bias and dependence on the accuracy of documented complications. Nonetheless, by including all consecutive cases over 3 years and ensuring robust follow-up, we were able to capture clinically relevant complications and port outcomes. The relatively large single-center sample size (737 patients) also ensured standardized techniques within each procedural setting. Second, we did not formally record minor complications (e.g., mild bruising and temporary pain) or patient-centered outcomes such as comfort and satisfaction, which may differ between settings (for instance, some patients might prefer mild sedation in OR vs. just local anesthesia in the IR). These outcomes were beyond the scope of our study but warrant further investigation. Finally, although we analyzed patient-related factors, such as BMI and diabetes, data on glycemic control, duration of diabetes, and diabetic complications were unavailable. These unmeasured variables may have influenced infection- or wound-related outcomes, introducing potential residual confounding.

## 5. Conclusions

Chemoport implantation can be safely performed in both IR and OR settings, with comparable rates of major and late complications. The findings support the use of the IR suite as a cost-effective and resource-efficient alternative to the OR without compromising patient safety or procedural quality.

## Figures and Tables

**Table 1:** Baseline demographics and clinical characteristics of patients in the IR and OR groups.

	IR (*n* = 544)	OR (*n* = 193)	Total (*n* = 737)	*p*-Value
Age (years)	67.6 ± 12.4	68.6 ± 13.1	67.8 ± 12.6	0.175
Sex, *n* (%)				
Male	283 (52.0%)	116 (60.1%)	399	0.053
Female	261 (48.0%)	77 (39.9%)	338	
DM history	96 (17.6%)	38 (19.7%)	134	0.211
BMI	22.9 ± 4.3	22.6 ± 4.1	22.8 ± 4.3	0.730
Approach				
Right IJV	468 (86.0%)	179 (92.7%)	647	0.001
Left IJV	76 (14.0%)	14 (7.3%)	90	

**Table 3:** Results of the univariate and multivariate Cox proportional hazards analysis for factors associated with complication occurrence.

Variables	Univariate Cox Regression Analysis			Multivariable Cox Regression Analysis		
	HR	95% CI	*p*	Adjusted HR	95% CI	*p*
IR vs. OR	1.465	0.770–2.788	0.244	1.557	0.802–3.020	0.191
Sex	1.026	0.558–1.884	0.935	0.986	0.514–1.890	0.966
Age	1.009	0.983–1.034	0.509	1.010	0.985–1.036	0.442
BMI	1.070	0.998–1.147	0.056	1.076	1.000–1.158	0.051
DM	1.165	0.800–1.698	0.426	1.095	0.713–1.683	0.678
Approach (Rt. vs. Lt.)	1.591	0.705–3.587	0.263	1.966	0.825–4.682	0.127

HR, hazard ratio; CI, confidence interval; BMI, body mass index; DM, diabetes mellitus.

## Data Availability

The dataset generated and/or analyzed during the current study is available from the corresponding author upon reasonable request.

## Abbreviations

IR, Interventional Radiology; OR, Operating Room; IJV, Internal Jugular Vein; DM, Diabetes Mellitus; BMI, Body Mass Index.
